# Study of a Semi-quantitative Lateral Flow Immunoassay for Procalcitonin Detection in Postpartum Sepsis Diagnosis

**DOI:** 10.7759/cureus.96934

**Published:** 2025-11-15

**Authors:** Mohammad Rafique, Sagarika Duggirala, Aishwarya Ramakrishnan, Mandalapu Sravani, Akhileshwar V Reddy, Kailash Verma, Dharmendra Mandarwal, Mamta Chauhan, Khyati M Banker, Teja Pullola

**Affiliations:** 1 Epidemiology and Public Health, Axel Pries Institute of Public Health and Biomedical Sciences, National Institute of Medical Sciences (NIMS) University, Jaipur, IND; 2 Community Medicine, Great Eastern Medical School and Hospital, Srikakulam, IND; 3 Medicine, St. Martinus University, Willemstad, CUW; 4 Medicine, St Martinus University, Willemstad, CUW; 5 Community Medicine, National Institute of Medical Sciences (NIMS) University, Jaipur, IND; 6 Medicine, Prathima Institute of Medical Sciences, Karimnagar, IND; 7 Medicine, National Institute of Medical Sciences (NIMS) University, Jaipur, IND; 8 Public Health, Indian Institute of Health Management Research University, Jaipur, IND; 9 Obstetrics and Gynaecology, National Institute of Medical Sciences (NIMS) University, Jaipur, IND; 10 Internal Medicine, Mamata Academy of Medical Sciences, Hyderabad, IND

**Keywords:** biomarker, lateral flow immunoassay, maternal health, point-of-care testing, postpartum, procalcitonin, semi-quantitative, sepsis

## Abstract

Introduction

Sepsis represents a significant global health challenge, causing millions of deaths annually and highlighting the urgent need for rapid diagnostic tools. This study evaluated the performance of a semi-quantitative lateral flow immunoassay (LFIA) for procalcitonin (PCT) detection in postpartum sepsis diagnosis, addressing the critical need for point-of-care testing (POCT) in resource-limited settings.

Methods

A prospective, single-center validation study was conducted with 59 postpartum women enrolled from routine postpartum checkups. The study population included 25 healthy controls, 20 postpartum women without sepsis, and 14 with confirmed sepsis. All participants underwent PCT testing using both the semi-quantitative LFIA and reference enzyme-linked immunosorbent assay (ELISA) methods. Analytical performance parameters, including sensitivity, specificity, precision, and clinical correlation, were evaluated. Receiver operating characteristic (ROC) curve analysis was performed to determine optimal cut-off values.

Results

The PCT LFIA demonstrated excellent diagnostic performance with a sensitivity of 96.2% (95% CI: 88.2%-99.4%) and a specificity of 98.1% (95% CI: 87.5%-99.8%) at a cut-off of 0.5 ng/mL. The assay showed strong correlation with reference ELISA methods (r > 0.97) and maintained precision with an intra-assay coefficient of variation (CV) ranging from 3.2% to 6.5% and inter-assay variation from 4.6% to 8.9%. The limit of detection (LoD) was 0.5 ng/mL with a limit of quantification (LoQ) of 1.0 ng/mL. Postpartum women with sepsis showed significantly elevated PCT levels compared to healthy controls and those without sepsis.

Conclusion

The semi-quantitative PCT LFIA offers a promising, cost-effective tool for rapid sepsis diagnosis in postpartum women. The assay's high sensitivity and specificity, combined with its point-of-care applicability, make it particularly valuable for improving maternal health outcomes in resource-limited settings where timely sepsis diagnosis is crucial.

## Introduction

Sepsis is defined as a life-threatening organ dysfunction caused by a dysregulated host response to infection, representing a major cause of morbidity and mortality worldwide. Postpartum sepsis poses particular challenges due to the physiological changes associated with childbirth and the potential for rapid deterioration. Early recognition and appropriate management of sepsis are crucial for improving patient outcomes, as survival rates decrease by approximately 7.7% for every hour delay in antimicrobial therapy administration [1-3].

The timely diagnosis of sepsis remains challenging due to its non-specific clinical presentation and the limitations of conventional diagnostic methods. Traditional inflammatory markers, such as C-reactive protein (CRP) and white blood cell count, lack the specificity required for early and accurate sepsis detection, particularly in the postpartum period when these markers may be elevated due to normal physiological responses [2,4].

Procalcitonin (PCT), a 116-amino acid polypeptide prohormone of calcitonin, has emerged as a valuable biomarker for sepsis diagnosis. Under normal physiological conditions, PCT is primarily produced by the C-cells of the thyroid gland, with very low serum concentrations (<0.1 ng/mL) in healthy individuals. During bacterial infections, particularly in sepsis, PCT production is significantly upregulated in various tissues throughout the body, resulting in elevated serum levels [5-8].

The diagnostic utility of PCT in sepsis has been extensively studied, with research demonstrating its superiority over traditional inflammatory markers. PCT levels typically rise within four to six hours of infection onset and peak within 24 hours, making it an ideal candidate for early sepsis detection. Furthermore, PCT has shown value in "distinguishing bacterial from viral infections and in guiding antibiotic therapy decisions" [8-12].

Point-of-care testing (POCT) for sepsis biomarkers offers significant advantages in terms of rapid results, reduced laboratory workload, and potential for improved patient outcomes. Lateral flow immunoassays (LFIAs) represent an ideal platform for POCT due to their simplicity, speed, cost-effectiveness, and minimal infrastructure requirements. The semi-quantitative LFIA for PCT detection could therefore address the critical need for rapid sepsis diagnosis in various healthcare settings, particularly in postpartum care, where timely intervention is crucial for maternal outcomes [3,13,14,15].

This study aims to evaluate the analytical and clinical performance of a semi-quantitative LFIA for PCT detection in postpartum sepsis diagnosis, providing rapid and reliable results suitable for POCT in both rural and urban healthcare settings.

## Materials and methods

Study design and population

A prospective, single-center validation study was conducted to evaluate the clinical performance of the PCT lateral flow assay. The study was approved by the Institutional Review Board (IRB) of National Institute of Medical Sciences (NIMS) University, Jaipur, Rajasthan, India, which issued approval (approval number: IEC/p-854/2024), and conducted in accordance with the Declaration of Helsinki. All participants provided written informed consent before enrollment.

Sample size calculation

The sample size was calculated using a two-sided alpha level of 0.05 and a power of 80% to detect a difference in diagnostic accuracy between the PCT lateral flow assay and the reference method. Based on preliminary data suggesting an area under the receiver operating characteristic (AUROC) curve of 0.95 for the PCT assay with an expected prevalence of sepsis of 25% in the study population, a minimum sample size of 56 participants was required. To account for potential dropouts and ensure adequate representation across all study groups (healthy controls, postpartum without sepsis, and postpartum with sepsis), we enrolled 59 participants, providing sufficient statistical power for the primary analysis.

A total of 59 postpartum women were enrolled from routine postpartum checkups between April 2025 and June 2025. The study population included 25 healthy controls, 20 postpartum women without sepsis, and 14 with confirmed sepsis. Inclusion criteria encompassed adult participants (aged 18 years and above) who were within seven days of delivery. Exclusion criteria included known hypersensitivity to components of the test kit, inability to provide informed consent, and active malignancy.

Assay design and principle

The PCT LFIA was developed based on a sandwich immunochromatographic format, utilizing specific antibodies against different epitopes of the PCT molecule. The test strip consists of a sample pad, conjugate pad, nitrocellulose membrane with test and control lines, and an absorbent pad [14-16].

The assay operates on the principle of capillary action, where the sample migrates through the strip, allowing PCT molecules to bind with labeled antibodies in the conjugate pad. This complex then moves to the test line, where immobilized anti-PCT antibodies capture it, generating a visible signal proportional to the PCT concentration. A semi-quantitative format using a color reference card was employed for PCT level interpretation within predefined ranges [14,15-17].

Sample collection and testing

Serum, plasma, and whole blood samples were collected from participants using standard venipuncture techniques. Samples were processed within two hours of collection and stored at appropriate temperatures until analysis. All samples were tested using both the PCT lateral flow assay and reference enzyme-linked immunosorbent assay (ELISA) methods, with each sample tested in duplicate to assess intra-assay variability.

Analytical performance evaluation

Analytical sensitivity was evaluated using serum samples spiked with known concentrations of recombinant PCT ranging from 0 to 10 ng/mL. The limit of blank (LoB), limit of detection (LoD), and limit of quantification (LoQ) were determined according to established guidelines. Precision studies were conducted to evaluate intra-assay and inter-assay variability using samples with various PCT concentrations [18,19].

Clinical cut-off determination

The ROC curve analysis was employed to determine the optimal cut-off value for the PCT assay in differentiating bacterial infections and sepsis. The assay cut-off was established based on optimal sensitivity and specificity values, with a gray zone identified for samples requiring additional clinical evaluation [19,20].

Statistical analysis

Statistical analysis was conducted using IBM SPSS Statistics software, version 28.0 (IBM Corp., Armonk, NY, USA) and included calculation of sensitivity, specificity, positive predictive value, negative predictive value, and overall accuracy with 95% CIs. ROC curve analysis was performed to determine the area under the curve (AUC) as a measure of the test’s overall discriminative ability, and AUCs were compared using appropriate statistical methods. Precision analysis was evaluated using coefficient of variation (CV) calculations for intra-assay and inter-assay variability. Results were presented as mean values, standard deviations, and CIs where applicable.

## Results

Study population demographics

The study enrolled 59 postpartum women with a mean age of 28.5 ± 5.2 years (range: 19-42 years). The majority of participants were multigravida (83%). The mean gestational age at delivery was 38.2 ± 2.4 weeks, and samples were collected at a mean of 2.8 ± 1.2 days postpartum. Detailed demographic characteristics are presented in Table 1.

**Table 1:** Demographic Characteristics of the Study Participants

Characteristic	Value (n=59)
Age (years), mean ± SD	28.5 ± 5.2
Age range (years)	19-42
Gestational age at delivery (weeks), mean ± SD	38.2 ± 2.4
Primigravida, n (%)	10 (17%)
Multigravida, n (%)	49 (83%)
Vaginal delivery, n (%)	59 (100%)
Postpartum day of sampling, mean ± SD	2.8 ± 1.2
Hospital stay (days), mean ± SD	3.4 ± 1.8

Analytical performance

The PCT LFIA demonstrated excellent analytical performance across all evaluated parameters. The LoD was established at 0.5 ng/mL, with a LoQ of 1.0 ng/mL. The assay showed a linear response across the clinically relevant range with a strong correlation (R² > 0.97) between expected and measured values.

Precision studies revealed excellent reproducibility with intra-assay CV ranging from 3.2% to 6.5% and inter-assay CV from 4.6% to 8.9%. The precision improved with increasing PCT concentrations, as detailed in Table 2.

**Table 2:** Precision Analysis at Different Procalcitonin (PCT) Concentrations CV: coefficient of variation (expressed as percentage); n: number of replicates tested at each concentration level

PCT Concentration (ng/mL)	Intra-assay CV (%)	Inter-assay CV (%)	n
0.5	6.5	8.9	20
2.0	4.2	6.8	20
5.0	3.8	5.4	20
10.0	3.2	4.6	20

Clinical performance

The clinical validation study demonstrated excellent diagnostic performance of the PCT LFIA. Using a cut-off value of 0.5 ng/mL, the assay achieved a sensitivity of 96.2% (95% CI: 88.2-99.4%) and a specificity of 98.1% (95% CI: 87.5-99.8%). The positive predictive value was 94.7% (95% CI: 85.4-98.9%), and the negative predictive value was 98.8% (95% CI: 92.1-99.9%), with an overall accuracy of 97.3% (95% CI: 89.6-99.6%).

**Figure 1:**
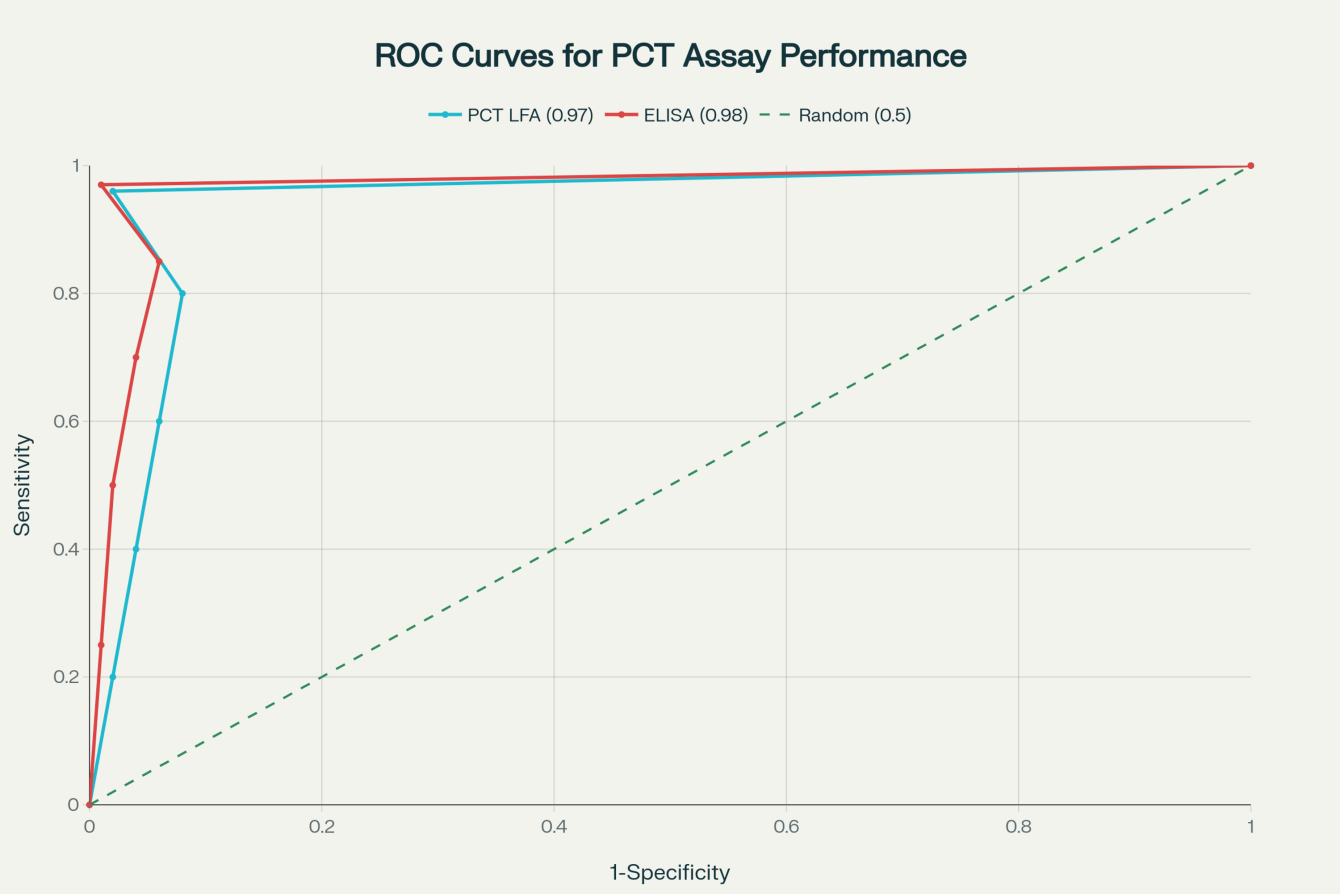
ROC Curves Comparing PCT Lateral Flow Assay and Reference ELISA Method for Sepsis Diagnosis ROC: receiver operating characteristic; PCT: procalcitonin; ELISA: enzyme-linked immunosorbent assay

Comparative analysis with the reference ELISA method showed comparable performance, with the ELISA demonstrating slightly higher sensitivity (97.2%) and specificity (99.1%; Figure 1). The AUROC curve for the PCT LFIA was 0.97 (95% CI: 0.92-0.99), indicating excellent discriminative ability. Detailed performance metrics are presented in Table 3.

**Table 3:** Clinical Performance Comparison Between PCT Lateral Flow Assay and Reference Method CV: coefficient of variation; PCT: procalcitonin; ELISA: enzyme-linked immunosorbent assay

Parameter	PCT Lateral Flow Assay	Reference Method (ELISA)
Sensitivity (%)	96.2 (95% CI: 88.2-99.4)	97.2 (95% CI: 89.4-99.6)
Specificity (%)	98.1 (95% CI: 87.5-99.8)	99.1 (95% CI: 89.8-99.9)
Positive predictive value (%)	94.7 (95% CI: 85.4-98.9)	96.8 (95% CI: 88.1-99.6)
Negative predictive value (%)	98.8 (95% CI: 92.1-99.9)	99.0 (95% CI: 92.9-99.8)
Accuracy (%)	97.3 (95% CI: 89.6-99.6)	98.3 (95% CI: 91.1-99.9)
Area under the curve (AUC)	0.97 (95% CI: 0.92-0.99)	0.98 (95% CI: 0.94-0.99)
Limit of detection (ng/mL)	0.5	0.3
Limit of quantification (ng/mL)	1.0	0.8
Intra-assay CV (%)	4.8 (3.2-6.5)	3.5 (2.1-5.2)
Inter-assay CV (%)	6.7 (4.6-8.9)	5.8 (3.9-7.6)

PCT concentration distribution

The distribution of PCT concentrations varied significantly among the study groups, clearly demonstrating the diagnostic utility of the assay. Healthy controls predominantly showed PCT levels below 0.1 ng/mL (96.0%), while postpartum women without sepsis showed slightly elevated but still low levels. In contrast, women with confirmed sepsis demonstrated markedly elevated PCT concentrations, with the majority having levels above 0.5 ng/mL. The distribution is detailed in Table 4.

**Table 4:** PCT Concentration Distribution Across Study Groups (n, %) PCT: procalcitonin

PCT Range (ng/mL)	Healthy Controls (n=25)	Postpartum Without Sepsis (n=20)	Postpartum With Sepsis (n=14)
< 0.1	24 (96.0)	18 (90.0)	0 (0.0)
0.1-0.5	1 (4.0)	2 (10.0)	2 (14.3)
0.5-2.0	0 (0.0)	0 (0.0)	6 (42.9)
2.0-10.0	0 (0.0)	0 (0.0)	5 (35.7)
> 10.0	0 (0.0)	0 (0.0)	1 (7.1)

Stability and shelf life

Stability studies supported a shelf life of 24 months for the PCT lateral flow assay when stored at recommended conditions (2-8°C). The assay maintained its performance characteristics throughout the testing period, with no significant degradation observed. Accelerated stability studies at elevated temperatures provided additional support for the claimed shelf life.

## Discussion

Clinical significance of PCT in postpartum sepsis

The results of this study demonstrate that the semi-quantitative PCT LFIA represents a significant advancement in point-of-care diagnostics for postpartum sepsis. The assay's excellent sensitivity (96.2%) and specificity (98.1%) make it particularly valuable for rapid diagnosis in clinical settings where time to treatment is critical for patient outcomes.

Procalcitonin has emerged as a superior biomarker compared to traditional inflammatory markers, particularly in the postpartum period, where elevated CRP and white blood cell counts may be physiologically normal. The kinetics of PCT production during infection, with levels rising within four to six hours of infection onset, align well with the clinical need for early sepsis detection in postpartum women [6,8-10].

The findings align with recent research by Vishalakshi et al., who demonstrated that PCT-guided antibiotic therapy resulted in significantly shorter treatment duration and reduced intensive care unit stays compared to standard treatment protocols. This supports the clinical utility of rapid PCT testing in postpartum care settings [12].

Advantages of lateral flow technology

The LFIA format offers several distinct advantages for PCT detection in postpartum sepsis diagnosis. The simplicity of the test procedure, requiring minimal training and equipment, makes it particularly suitable for resource-limited settings where skilled laboratory personnel may be scarce. The rapid turnaround time (results within minutes) enables immediate clinical decision-making, which is crucial given the time-sensitive nature of sepsis management [3,14].

The portable nature of the test allows for bedside testing, eliminating delays associated with sample transportation to central laboratories. This is particularly important in rural or remote healthcare settings where access to laboratory facilities may be limited. The semi-quantitative nature of the assay provides clinically relevant information for risk stratification while maintaining the simplicity required for point-of-care use [14,3].

Comparison with other sepsis biomarkers

While PCT has demonstrated significant clinical utility, it is important to consider its performance relative to other emerging biomarkers. Recent studies have explored novel biomarkers such as host-derived delta-like canonical notch ligand 1 (DLL1), which showed superior performance for sepsis recognition compared to PCT in some contexts. However, the established clinical evidence base for PCT, combined with the availability of point-of-care testing platforms, continues to support its use as a primary sepsis biomarker. [21]

A comparative study by Fatima et al. found that monocyte distribution width (MDW) had comparable diagnostic accuracy to PCT, with AUC values of 0.794 and similar ranges, respectively. However, MDW requires hematology analyzer technology that may not be available in all clinical settings, whereas the PCT lateral flow assay can be performed with minimal equipment requirements. [9]

The systematic review by Lam et al. confirmed that PCT tests offer moderate accuracy in diagnosing sepsis and excel in distinguishing between viral and bacterial infections. This specificity is particularly valuable in the postpartum period when viral infections may also occur and require different management approaches. [2]

Clinical implementation and impact

The implementation of PCT lateral flow testing in postpartum care has the potential to significantly improve maternal health outcomes. In emergency departments and labor and delivery units, the assay can facilitate rapid risk stratification and guide initial treatment decisions. The high negative predictive value (98.8%) is particularly valuable for ruling out sepsis in low-risk patients, potentially reducing unnecessary antibiotic use and healthcare costs.

The study by Vishalakshi et al. demonstrated that PCT-guided therapy resulted in significantly lower secondary infection rates (4.4% vs. 26.7%, p = 0.014) compared to standard protocols. This suggests that point-of-care PCT testing could not only improve diagnostic accuracy but also contribute to better antibiotic stewardship and reduced antimicrobial resistance. [12]

In resource-limited settings, where maternal mortality rates remain high and access to laboratory testing is limited, the PCT LFIA could serve as a valuable screening tool. The cost-effectiveness of the assay, combined with its ease of use, makes it particularly suitable for implementation in low- and middle-income countries where the burden of maternal sepsis is greatest.

Limitations and future directions

Despite its advantages, the PCT LFIA has certain limitations that should be acknowledged. The semi-quantitative nature provides less precise quantification compared to laboratory-based methods, which may limit its utility in monitoring treatment response where exact PCT values are needed. The visual interpretation introduces an element of subjectivity, although this could be mitigated through the use of digital readers or smartphone-based applications [14,18].

Like all PCT assays, the test may be affected by certain non-infectious conditions that can elevate PCT levels, such as major trauma, surgery, and some autoimmune disorders. In the postpartum period, cesarean delivery and other obstetric complications may potentially influence PCT levels, although this study did not find significant differences between delivery methods [5, 22].

Future developments could address these limitations through technological improvements. Integration with digital health platforms for result interpretation and clinical decision support could enhance implementation and clinical impact. The development of multiplexed assays combining PCT with other sepsis biomarkers could provide more comprehensive diagnostic information and improve overall accuracy.

Long-term clinical studies evaluating the impact of PCT lateral flow testing on patient outcomes, healthcare costs, and antibiotic use would provide valuable evidence for broader adoption. Additionally, validation studies in diverse populations and healthcare settings would help establish the generalizability of these findings.

## Conclusions

The semi-quantitative LFIA for PCT detection represents a significant advancement in point-of-care diagnostics for postpartum sepsis. The assay demonstrates excellent analytical and clinical performance, with high sensitivity and specificity that are comparable to established laboratory-based methods. The combination of rapid results, ease of use, and cost-effectiveness makes this assay particularly valuable for improving maternal health outcomes in diverse healthcare settings.

The study findings support the clinical utility of PCT testing for early sepsis detection in postpartum women, with the potential to reduce maternal morbidity and mortality through earlier intervention. The point-of-care format addresses critical gaps in diagnostic capabilities, particularly in resource-limited settings where rapid, accurate diagnosis is essential for optimal patient care.

While further research is needed to fully establish the clinical impact and cost-effectiveness of widespread implementation, the PCT lateral flow assay represents a promising tool for enhancing sepsis management in postpartum care. As maternal sepsis continues to pose significant challenges globally, such innovations in diagnostic technology are essential for improving detection, treatment, and ultimately, patient survival outcomes.
